# Intraosseous hibernoma: case report and tumor characterization

**DOI:** 10.1259/bjrcr.20150204

**Published:** 2015-07-29

**Authors:** A Jerman, Ž Snoj, B G Kuzmanov, A K Limpel Novak

**Affiliations:** ^1^ Faculty of Medicine, University of Ljubljana, Ljubljana, Slovenia; ^2^ Radiology Department, University Medical Centre Ljubljana, Ljubljana, Slovenia; ^3^ Pathology Department, Institute of Oncology, Ljubljana, Slovenia

## Abstract

Intraosseous hibernoma is a rare finding that has only recently come to light in the literature. We report a case of intraosseous hibernoma in the sacrum identified as an incidental finding in a 40 year-old female with chronic lower back pain. The tumor was characterized with all routine imaging modalities. In the review of the literature we correlate the imaging findings with previously reported cases. With increasing number of radiologic investigations it is expected to discover more intraosseous hibernomas and the radiologists should consider intraosseous hibernoma as differential diagnosis of the sclerotic bone lesion.

## Summary

Hibernoma is a benign lipomatous tumour composed of the brown adipose cells. It was first described by Merkel^1^ in 1906 as pseudolipoma and renamed hibernoma by Gery^2^ in 1914.^3^ In adults, the brown adipose tissue is found in the upper trunk, paravertebral space and around major arteries.^4^ It has an important role in thermoregulation, development of obesity and insulin sensitivity in humans as an antidiabetic tissue.^5,6^ Soft-tissue hibernoma is an uncommon tumour and is predominantly found in the subcutaneous and muscle tissue of the hips and the upper trunk. However, intraosseous hibernoma is a rare finding that has only recently come to light in the literature.

We report a case of an intraosseous hibernoma found as an incidental finding in the sacrum of a 40-year-old Caucasian female with chronic lower back pain. All routine imaging modalities were performed to characterize the tumour. In the review of the literature, we correlate the imaging findings with previously reported cases. Furthermore, we show that the intraosseous hibernoma has similar features on all routine imaging modalities, except on post-contrast MRI, where the most common pattern appears to be a moderate enhancement throughout the tumour and in the peripheral rim.

## Case report

**Figure 1.**
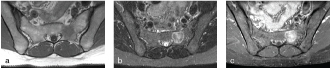
MRI of the pelvis. (a) Axial *T*_1_ weighted image showing a 21-mm round, homogeneous lesion of low signal intensity interforaminally in the left S2 segment of the sacrum. (b) Axial short tau inversion-recovery image showing a well-defined lesion with inhomogeneous signal intensity throughout the lesion with high peripheral rim intensity. (c) Axial *T*_1_ image with fat saturation after contrast media injection showing moderate enhancement throughout the lesion and in the peripheral rim.

**Figure 2.**
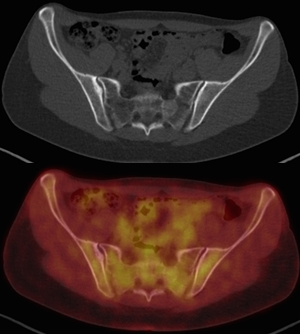
PET-CT scan showing a well-defined sclerotic lesion with mild fludeoxyglucose avidity (average standardized value 2.5) in the S2 segment. No other abnormalities were found on PET-CT scan. PET, positron emission tomography.

**Figure 3.**
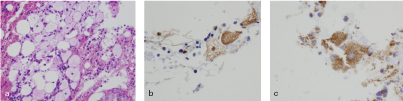
Histopathological images. (a) An infiltration of the otherwise normal bone marrow with scattered small groups of big foamy cells. (b) Immunohistochemistry shows lesional cells expressing S100 protein, indicative of fatty infiltration. (c) The cells contain multiple lipid droplets and numerous large mitochondria, shown with antimitochondrial marker.

In a work-up of a 40-year-old female with a history of low back pain, a routine MRI of the lumbar spine identified an incidental lesion. The axial *T*_1_ weighted image showed a 21-mm round, homogeneous lesion of low signal intensity interforaminally in the left S2 segment of the sacrum. The axial short tau inversion-recovery image showed a well-defined lesion with an inhomogeneous signal intensity throughout the lesion with high peripheral rim intensity. The axial *T*_1_ image with fat saturation after contrast media injection showed moderate enhancement throughout the lesion and in the peripheral rim (Figure 1). A radiograph of the pelvis was performed to further characterize the lesion. On clinical examination, the sacral area was not painful to palpation. Owing to the atypical appearance of the lesion and the non-specific nature of the MRI signal pattern, a bone scan was performed that showed a solitary lesion on the left side of the sacrum with increased metabolic activity. For further characterization, and in the search of a potential primary tumour, positron emission tomography (PET)-CT was performed, which showed a well-defined sclerotic lesion with mild fludeoxyglucose (FDG) avidity (average standardized value 2.5) in the S2 segment and no other abnormalities (Figure 2). Owing to the metabolic activity of the lesion, the patient was referred to the orthopedic oncology department for image-guided biopsy of the lesion. The biopsy specimen consisted of a few small fragments of bone marrow, some skeletal muscle, fibroadipose tissue and blood clots. Infiltration of the otherwise normal bone marrow with scattered small groups of big foamy cells was identified (Figure 3a). The foamy cells had vacuolated cytoplasm and small centrally located nuclei. The cells were negative for cytokeratin AE1/AE3, CD68, barchyury, Melan A, HMB 45, desmin and smooth muscle actin but positive for S100 protein (Figure 3b). The cells contained multiple lipid droplets and numerous large mitochondria; the existence of the latter was exhibited with antimitochondrial marker (Figure 3c). A pathological diagnosis of hibernoma was made in correlation with the imaging findings.

Owing to the clinical assessment in correlation with the history of bilateral lumbago, worse after physical activity, the patient was diagnosed with chronic lower back pain. At 1-year follow-up, the patient was being managed with analgesics and physical therapy.

## Discussion

We present a case of a rare intraosseous benign brown adipose tissue tumour in the sacrum with imaging characterization of this rare neoplasm. Furthermore, we review the imaging findings in conjunction with the previously reported cases.

Hibernomas are benign neoplastic tumours composed of brown fat. Soft-tissue hibernomas represent 1.6% of all benign lipomatous tumours and approximately 1.1% of all adipocytic tumours.^7^ They are usually located in the thighs, shoulders, back and neck; however, their location vary.^8^ The peak incidence of soft-tissue hibernomas is in the fourth decade and it is more common in men.^8^ In comparison with soft-tissue hibernomas, intraosseous hibernomas are a rare finding. The Medline search revealed 7 papers describing 11 cases of intraosseous hibernoma since 2008 (Table 1). Intraosseous hibernomas are mostly found in the axial skeleton and are asymptomatic. One case was considered to be symptomatic as the symptoms resolved 9 months after percutaneous radiofrequency ablation of the lesion.^14^

**Table 1.** Previously published cases of intraosseous hibernoma.

Case Report	Age (years)	Sex	Location	Imaging modality		Year published
1	61	F	Ilium	None	Thorns et al^9^	2008
2	57	M	Sacrum	MR, CT	Kumar et al^10^	2011
3	77	F	Iliac crest	None	Lynch et al^11^	2013
4	50	F	Ilium	MR, PET-CT	Bai et al^12^	2013
5	40	F	Posterior ilium	MR, CT, bone scan	Botchu et al^13^	2013
6	70	F	Left sacrum	MR, CT	Ringe et al^14^	2013
7	48	F	T5 vertebral body	MR, CT, PET scan	Bonar et al^3^	2014
8	64	M	Manubrium sterni	Bone scan, SPECT-CT	Bonar et al^3^	2014
9	71	M	Ischiopubic ramus	CT, bone scan, SPECT-CT, X-ray	Bonar et al^3^	2014
10	50	F	T12 vertebral body	MR, CT, bone scan, PET scan	Bonar et al^3^	2014
11	85	M	Left iliac crest	CT	Bonar et al^3^	2014
12	40	F	Left sacrum	MR, bone scan, PET-CT, X-ray	Our case	2015

F, female; M, male; PET, positron emission tomography; SPECT, single photon emission computed tomography.

In order to rule out other tumours in the differential diagnoses of sclerotic bone lesions (metastases, intraosseous haemangioma, bone island, lymphoma and notochordal rest) and make a final diagnosis, a biopsy needs to be performed. Typical histopathological findings include multivacuolated, foamy fat cells with small eccentric nuclei that do not show any features of malignancy. Foamy cells are distributed in small groups among the thickened bone trabecula where they replace the normally present white fat and bone marrow cells. Immunohistochemical investigations have revealed that the foamy cells are positive for S100 protein and negative for cytokeratin AE1/AE3, CD68 and barchyury.^3^ These are characteristic findings of brown fat cells, and it is noteworthy that cells are negative for these markers in order to exclude chondroma and histiocytic lesions.^3^

All routine imaging modalities were performed on our patient to try and characterize the lesion. In only one previous case had a plain radiograph been taken and it showed a well-defined sclerotic lesion in the left ischiopubic ramus. However, no lesion could be identified on the plain radiograph of our patient. This can be attributed to the location of the lesion in the sacrum, with overprojecting surrounding structures. The tumour was sclerotic on the CT scan of our patient and all the previously reported cases (Table 2). The tumour showed moderately increased metabolic activity on the bone scan, whereas on the PET scan, the tumour showed mild FDG avidity. Both findings are consistent with the previously reported cases. On MRI, the tumour showed characteristics similar to the previously reported cases—*T*_1_ hypointense to the subcutaneous fat and *T*_2_ hyperintense to the skeletal muscle. However, in correlation with previous reports, the reported lesions show different characteristics of enhancement on post-contrast MRI. We divided the post-contrast MRI characteristics into three groups (Table 2). In our case, after the application of paramagnetic contrast, the lesion showed characteristics similar to the two previously reported cases, with moderate enhancement throughout the lesion and in the peripheral rim.^3,14^ In other reports, after the application of paramagnetic contrast, the tumour showed enhancement throughout the lesion without enhancing peripheral rim (one case) or the lesion showed no enhancement (one case; Table 2).^10,12^ No lesion showed overt aggressive features on imaging.

**Table 2.** Imaging findings in previously published cases of intraosseous hibernoma including our patient (where diagnostic imaging was performed).

Modality	Number	Findings
Plain film	2	Sclerosis (1/2) No pathological finding (1/2)
CT	10	Sclerosis in all
MR	7	*T*_1_ hypointense to subcutaneous fat and hyperintense to skeletal muscle (7/7) *T*_2_ hyperintense with high signal rim (6/7) Performed contrast enhancement (5/7) No enhancement (1/5) Enhancement throughout the lesion (1/5) Enhancement throughout the lesion with rim (3/5)
Bone scan	6	Minimal uptake (2/6) Pronounced uptake (4/6)
FDG PET	3	Mildly increased SUV (2.5, 3.0 and 3.3)

FDG, fludeoxyglucose; SUV, standardized uptake value.

Thorns et al^9^ were the first to describe a case of incidental finding of brown fat tissue in the bone marrow during a biopsy in 2008. Although only six articles have been published since then, the recent report by Bonar et al,^3^ with a case series of five intraosseous hibernomas, suggests that the tumour might not be as rare as originally thought. In the recent years, PET scans have identified that the presence of brown fat is more frequent than previously thought.^5^ Bonar et al^3^ even suggested the possibility that intraosseous brown tissue is a physiological phenomenon and proposed that the term “intraosseous brown-fat associated sclerosis” should be used instead of “intraosseous hibernoma”. Further studies are needed to confirm this; however, with the increasing number of radiological investigations, we can expect more cases of intraosseous hibernoma being discovered. Bearing this in mind, we concur with Bonar et al^3^ that this diagnosis should be added to the differential diagnosis of a sclerotic bone lesion.

## Learning points

1. Intraosseous hibernoma is a rare benign intraosseous neoplasm composed of brown tissue.
2. The majority of intraosseous hibernomas are discovered as an incidental finding.
3. The appearance of an intraosseous hibernoma on the CT scan is similar to a sclerotic bone lesion.
4. Intraosseous hibernoma has similar features on all routine imaging modalities except on post-contrast MRI, where the most common pattern appears to be a moderate enhancement throughout the tumour with an enhancing peripheral rim.
5. For immunohistochemical confirmation of intraosseous hibernoma, it is critical that brown fat cells test positive for S100 protein and negative for cytokeratin AE1/AE3, CD68 and barchyury.
